# Evaluation of Immunomodulatory Activities of the Heat-Killed Probiotic Strain *Lactobacillus casei* IMAU60214 on Macrophages In Vitro

**DOI:** 10.3390/microorganisms8010079

**Published:** 2020-01-07

**Authors:** Luz María Rocha-Ramírez, Beatriz Hernández-Ochoa, Saúl Gómez-Manzo, Jaime Marcial-Quino, Noemí Cárdenas-Rodríguez, Sara Centeno-Leija, Mariano García-Garibay

**Affiliations:** 1 Unidad de Investigación en Enfermedades Infecciosas, Hospital Infantil de México Federico Gómez, Secretaría de Salud Dr. Márquez No. 162, Col Doctores, Delegación Cuauhtémoc, Ciudad de México 06720, Mexico; 2 Laboratorio de Inmunoquímica y Biología Celular, Hospital Infantil de México Federico Gómez, Secretaría de Salud. Dr. Márquez No. 162, Col Doctores, Delegación Cuauhtémoc, Ciudad de México 06720, Mexico; beatrizhb_16@hotmail.com; 3 Laboratorio de Bioquímica Genética, Instituto Nacional de Pediatría, Secretaria de Salud, Ciudad de México 04530, Mexico; saulmanzo@ciencias.unam.mx; 4 Consejo Nacional de Ciencia y Tecnología (CONACYT), Instituto Nacional de Pediatría, Secretaría de Salud, Ciudad de México 04530, Mexico; jmarcialq@ciencias.unam.mx; 5 Laboratorio de Neurociencias, Instituto Nacional de Pediatría, Secretaría de Salud, Ciudad de México 04530, Mexico; noemicr2001@yahoo.com.mx; 6 Consejo Nacional Ciencia y Tecnologia (CONACYT) Laboratorio de Agrobiotecnología, Tecnoparque CLQ, Universidad de Colima, Carretera Los Limones-Loma de Juárez, Colima 28629, Mexico; scenteno0@ucol.mx; 7 Departamento de Ciencias de la Alimentación, Unidad Lerma, Universidad Autónoma Metropolitana, Av. San Rafael Atlixco No. 186. Col Vicentina, Ciudad de México 09340, Mexico; jmgg@xanum.uam.mx

**Keywords:** probiotic, *Lactobacillus casei* IMAU60214, health, innate immunity, macrophage, Toll-like receptors

## Abstract

Most *Lactobacillus* species have beneficial immunological (“immunoprobiotic”) effects in the host. However, it is unclear how probiotic bacteria regulate immune responses. The present study investigated the effects of heat-killed *Lactobacillus casei* IMAU60214 on the activity of human monocyte-derived macrophages (MDMs). Human MDMs were treated with heat-killed *L. casei* at a ratio (bacteria/MDM) of 50:1, 100:1, 250:1, and 500:1, and then evaluated for the following: NO production, by Griess reaction; phagocytosis of FITC-labeled *Staphylococcus aureus* particles; cytokine secretion profile (tumor necrosis factor (TNF)-α, interleukin (IL)-1β, IL-6, IL-12p70, IL-10, and transforming growth factor (TGF)-β) by ELISA; and costimulatory molecule (CD80 and CD86) surface expression, by flow cytometry. Heat-killed *L. casei* IMAU60214 enhanced phagocytosis, NO production, cytokine release, and surface expression of CD80 and CD86 in a dose-dependent manner. All products were previously suppressed by pretreatment with a Toll-like receptor 2 (TLR2)-neutralizing antibody. Overall, our findings suggest that this probiotic strain promotes an M1-like pro-inflammatory phenotype through the TLR2 signaling pathway. These effects on macrophage phenotype help explain the probiotic efficacy of *Lactobacillus* and provide important information for the selection of therapeutic targets and treatments compatible with the immunological characteristics of this probiotic strain.

## 1. Introduction

Lactic acid bacteria (LAB) constitute a heterogeneous group of Gram-positive, non-toxigenic facultative anaerobic microorganisms that efficiently produce lactic acid from carbohydrates, which makes them useful as starter cultures for food fermentation [1]. The most known LAB are the *Lactobacillus*, a genus that comprises a large heterogeneous group of LAB with more than 200 species and 29 subspecies of which more than 20 have been sequenced [1,2]. Among these is the *Lactobacillus casei* group (LCG), including *L. casei* and related species *L. paracasei* and *L. rhamnosus* [3]. The LCG group are the most extensively studied *Lactobacillus* species with documented health benefits [3,4] such as enhanced resistance against infection by increasing the secretion of pro-inflammatory cytokines (e.g., tumor necrosis factor (TNF)-α, interleukin (IL)-1β, and IL-12) from various immune cells analyzed on models of in vitro and in vivo investigations [5,6,7,8]. Multiple investigations have reported that treatment with probiotic bacteria increases resistance against pathogens that cause gastrointestinal and respiratory infections [9,10]. Furthermore, probiotic strains (both viable and non-viable), as well as specific cell wall components (e.g., peptidoglycans (PGs), lipoteichoic acids (LTAs), proteins, and exopolysaccharides (EPSs)) have the ability to stimulate the immune response [11,12]. In addition, the regions of these molecules exposed on the surface are considered microbe-associated molecular patterns (MAMPs), which can activate innate immune responses by binding to pattern recognition receptors (PRRs) expressed on immune cells and many other cell types [13,14]. Several reports have shown that the immunostimulant effect of the organism, in response to probiotics, is dependent in part on activation of Toll-like receptors (TLRs) [15,16]. Monocytes and macrophages express high levels of TLRs, mainly TLR2, TLR4, and TLR6, which have been demonstrated to induce cytokine and chemokine production upon stimulation by several probiotic species and strains [17,18,19]. Macrophages also participate in the activation and regulation of the immune response through antitumor activities, antigen presentation, and secretion of both pro-inflammatory cytokines (e.g., TNF-α, IL-1, and IL-6) and anti-inflammatory cytokines (IL-10 and transforming growth factor (TGF)-β) that act to regulate immune homeostasis [20,21,22]. In addition, probiotics act as mediators of inflammation and play an important role in the control of infection of several pathogens [23,24]. Several recent reports have examined the regulation of the macrophage immune response to probiotic strains of *Lactobacillus casei* [25]. However, the immune effects of individual probiotic strains cannot be generalized as each species of probiotic strain possesses unique functional properties and it is also important to consider that their effects are dependent on the specific host’s conditions [26,27]. Previously, we reported a screening study on some strains of *Lactobacillus* isolated from different fermented milks. Secretion profiles of inflammatory mediators such as cytokines varied depending on the *Lactobacillus* species present in the fermented milk [28].

In the present study, we extend the research on the isolate of *L. casei* IMAU60214, which has not been as extensively studied and is less characterized in comparison to others strains of probiotics, such as *L. rhamnosus* (LGG) [29]. Therefore, the aim of this study was to evaluate the immunomodulation activity in order to understand its probiotic potential and the role of microbial homeostasis in health and disease.

## 2. Materials and Methods

### 2.1. Bacterial Culture and Preparation of Heat-Killed Probiotic Bacteria

Strain *L. casei* IMAU60214 for this study was kindly provided by Dr. Guerrero from the fermented milk (Universidad Autonoma, Metropolitana, México). The *L. casei* IMAU60214 strain was grown in MRS broth (de Man, Ragosa, and Sharp Broth; BDL, Franklin Lakes, NJ, USA) at 37 °C for 18–24 h. Bacterial cells were harvested by centrifugation, and washed three times in physiological saline. The number of colony-forming units (CFUs) was determined by cell counting in dilutions of base 10. The concentration was adjusted to 1 × 10^9^ CFU and heat-killed at 85 °C for 10 min.

### 2.2. Preparation and Treatment of Human Monocyte-Derived Macrophages (MDMs)

Human peripheral blood mononuclear cells were obtained by gradient centrifugation using Lymphoprep (Nycomed, Zurich, Switzerland). Blood sampling, MDM isolation, and subsequent experiments were conducted with prior authorization and consent according to established institutional guidelines. Monocytes from healthy donors were enriched using the Monocyte Separation Kit II (Miltenyi Biotec, Bergisch Gladbach, Germany) and plated at 1 × 10^5^ cells per well in 24-well plates in RPMI-1640 medium supplemented with 10% fetal bovine serum (FBS) at 37 °C under a 5% CO_2_ atmosphere. Differentiated macrophages were obtained after seven days in culture. The purity after these cells was evaluated for phenotypic markers, CD14 and HLA-DR molecules (Biosciences, San Diego, CA, USA), determined by flow cytometry. Cells were then stimulated with heat-sensitive *L. casei* IMAU60214 at a ratio 50:1, 100:1, 250:1, and 500:1 of (bacteria/MDM) as indicated for 24 h. The research procedures in our study were approved by the Ethics Committee in Research (HIM-2014-013) of the Children′s Hospital, México and project code is HIM-2014-013, 1120 SSA (16 December 2014).

### 2.3. NO Production Assay

Nitric oxide (NO) production was assessed by quantifying the concentration of nitrite compounds (end-products of NO) in supernatants using a colorimetric assay based on the Griess reaction. Supernatants were recovered from untreated and treated MDM cells for 24 h with heat-killed *L. casei* IMAU60214, lipopolysaccharide (LPS) (1μg/mL) alone, LPS with polymyxin B (PMB) (1μg/mL) alone, the TLR2 agonist Pam3CSK4 (1μg/mL), or the TLR2 agonist with polymyxin B, respectively, and mixed with equal volumes of the Griess reagent (0.1% sulfanilamide, 5% phosphoric acid, and 1% ethylenediamine dihydrochloride). The mixture was incubated for 10 min at room temperature and the optical density measured at 540 nm. Nitrite concentration was obtained by extrapolation to a sodium nitrite standard curve.

### 2.4. Quantification of Cytokine Release

Cytokine levels (TNF-α, IL-1β, IL-6, IL-12p70, IL-10, and TGF-β) were measured in supernatants of treated and untreated MDMs using specific ELISA kits (BD Bioscience, San Diego, CA, USA) according to the manufacturer’s instructions.

### 2.5. Flow Cytometric Assay for Surface Marker Expression

Briefly, macrophages were treated with heat-killed *L. casei* IMAU60214 at ratio 500:1 (bacteria/MDM) at 37 °C under a 5% CO_2_ atmosphere for 24 h. Cells were harvested, washed twice with a 2 mM PBS/EDTA (pH 7.2) solution for 5 min, harvested, centrifuged in PBS (300× *g* for 5 min, 4 °C), resuspended in a blocking buffer solution (PBS supplemented with 2% FBS, 5 mM EDTA, human IgG immunoglobulin, and 0.1% sodium azide), and placed on ice for 30 min. Cells were centrifuged (300× *g* for 5 min), washed twice with PBS, and incubated for 30 min at 4 °C with specific markers in staining buffer (PBS supplemented with 2% FBS, 5 mM EDTA, and 0.1% sodium azide) containing FITC-conjugated anti-human CD80 and PE-conjugated anti-human CD86 (BD Bioscience, San Diego, CA, USA). Subsequently, cells were washed with 250 μL of staining solution and fixed with 2% paraformaldehyde in PBS containing 0.1% sodium azide. Isotype controls were used to estimate non-specific staining. In this study, we used PE-conjugated anti-IgG2a and FITC-conjugated IgG1 (Santa Cruz Biotechnology, Dallas, TX, USA). The proportions of cells expressing each marker as well as the average fluorescence value were acquired using a FACS Calibur flow cytometer (Becton Dickinson, Franklin Lakes, NJ, USA). Data were analyzed using WinMDI 2.9 software (manufacturer, city, state abbreviation, country). Results are expressed as mean fluorescence intensity (MFI) relative to control isotype for four independent experiments.

### 2.6. Phagocytosis Assay

Macrophages were pretreated with heat-killed *L. casei* IMAU60214 at a ratio 250:1 of (bacteria/MDM) for 24 h. A suspension containing FITC-labeled *Staphylococcus aureus* was added at a ratio 10:1 of (bacteria/MDM) and incubated for 1 h. The number of engulfed particles was visualized under a Zeiss Axiocam epifluorescence microscope (Zeiss AG, Oberkochen, Germany). The proportion of phagocytic macrophages and the number of engulfed particles were counted in randomly selected fields containing 60 cells.

### 2.7. Assays with a Toll-Like Receptor 2 (TLR2)-Neutralizing Antibody

The contributions of TLR2-dependent signaling to macrophage effector functions induced by heat-killed *L. casei* IMAU60214 at a ratio 500:1 of (bacteria/MDM) were analyzed by repeating these experiments in the presence of a TLR2-neutralizing antibody (TLR2 Bioscience clone TL 2.1, city, state abbreviation, country). Briefly, MDMs were treated with 10 μg/mL neutralizing antibody for 1 h at 37 °C followed by stimulation with probiotic bacteria for 24 hours as indicated.

### 2.8. Statistical Analysis

Data are presented as a mean ± standard deviation (SD); each experiment was performed with four donors and was performed in duplicate. Differences for nitric oxide and cytokine measurements between experimental groups (treated vs. untreated) were compared by Mann–Whitney U test. A *p* < 0.05 (two-tailed) was considered for statistical significance. Mean fluorescence intensity differences were shown as histograms.

## 3. Results

### 3.1. Purity and Morphological Identification of MDM Cells

**Figure 1:**
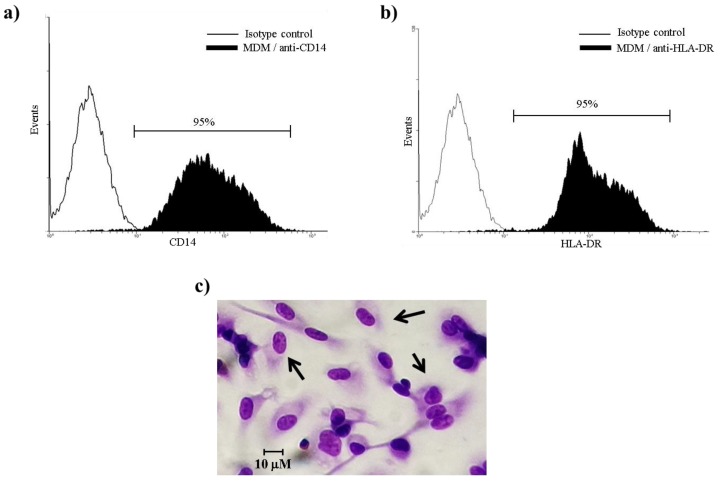
Purity and morphological identification of monocyte-derived macrophages (MDMs). CD14 and HLA-DR surface expression on MDM cells were evaluated after seven days in culture. (**a**) MDM cells were stained with isotype control Ab (IgG2) or (**b**) isotype control antibody (IgG1) and anti-HLA-DR-PE, and (**c**) morphology was identified by standard Giemsa staining. Results are from four healthy donors analyzed.

CD14 and HLA-DR expression were used as phenotypic markers for MDM cells during the differentiation process. FACS analysis of gated cells showed that 95% ± 2.9% expressed CD14 (Figure 1a) and a similar percentage was observed in the cells positive for HLA-DR (Figure 1b). Additionally, the purity of MDM cell was demonstrated by its morphology (Figure 1c).

### 3.2. Induction of NO Release from MDMs by Heat-Killed L. casei IMAU60214

**Figure 2:**
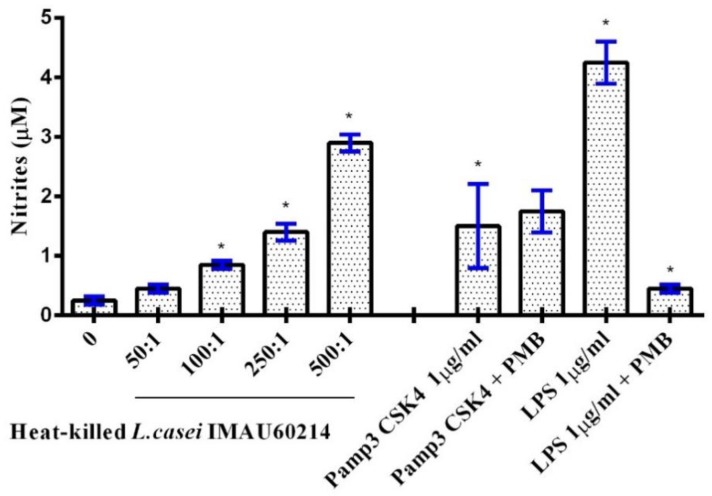
Dose-dependent effect of heat-killed *Lactobacillus casei* IMAU60214 on nitrite (nitric oxide (NO)) release by MDMs. Cells were cultured in duplicate in 96-well plates in the absence or presence of increasing heat-killed *L. casei* IMAU60214 at ratios of (bacteria/MDM) 50:1, 100:1, 250:1, and 500:1 for 24 h. After incubation, NO release was measured as described in the Materials and Methods section. Each value represents the mean ± SD of four independent experiments. * *p* < 0.05 was considered for statistically significant data compared to untreated controls.

The treatment of MDMs with heat-killed *L. casei* IMAU60214 at a ratio (bacteria/MDM) of 100:1, 250:1, and 500:1 for 24 h induced NO level elevation compared to the control negative (Figure 2). The highest induction was observed with (500:1, 250:1, 100:1) under these experimental conditions. In addition, the stimulatory activity of heat-killed *L. casei* IMAU60214 also revealed a dose-dependent increase in extracellular nitrate accumulation, a biochemical measure of NO release. Exposure to *Escherichia coli* 0111:B4 lipopolysaccharide (LPS, 1 μg/mL) induced NO release that was substantially greater than that produced by the probiotic heat-killed bacteria. However, the treatment with polymyxin B (an LPS inhibitor) effectively inhibited the NO production induced by LPS but had no effect on Pamp3CK4-induced NO production, indicating that enhanced NO production by *L. casei* IMAU60214 was not the result of endotoxin contamination.

### 3.3. Induction of Cytokine Release from MDMs by Heat-Killed L. casei IMAU60214

**Figure 3:**
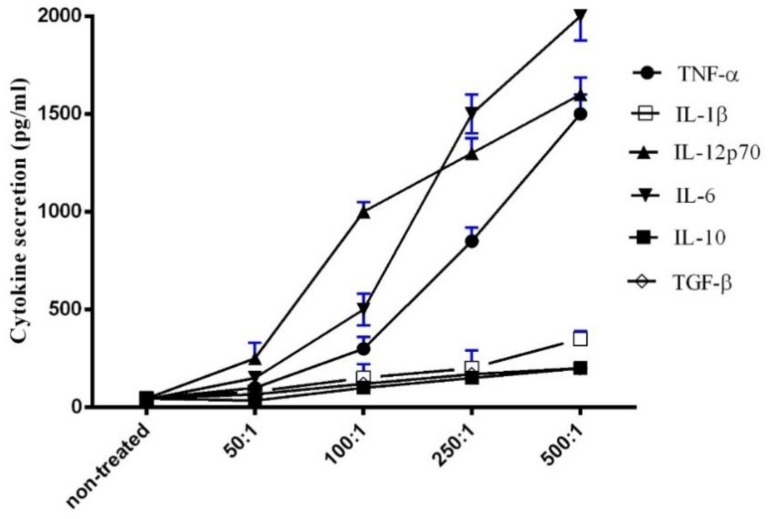
Heat-killed *L. casei* IMAU60214 dose-dependently enhances pro- and anti-inflammatory cytokine production by MDMs. The effects of *L. casei* IMAU60214 exposed at various ratios (50:1, 100:1, 250:1, 500:1) for 24 h on cytokine release were assessed by ELISA. Tumor necrosis factor (TNF)-α, interleukin (IL)-1β, IL-6, IL-12p70, IL-10, and transforming growth factor (TGF)-β. Each value represents the mean ± SD of four independent experiments.

The treatment with heat-killed *L. casei* IMAU60214 dose-dependently induced secretion of TNF-α, IL-1β, IL-6, IL-12p70, IL-10, and TGF-β with substantially greater extracellular accumulation at a ratio (bacteria/MDM) of 250:1 and 500:1 compared to 100:1 and 50:1 (Figure 3). In addition, accumulations of pro-inflammatory cytokines were higher than accumulations of the anti-inflammatory factors IL-10 and TGF-β, suggesting a shift toward pro-inflammatory status.

### 3.4. Induction of CD80 and CD86 Surface Expression by MDMs In Response to Heat-Killed L. casei IMAU60214

**Figure 4:**
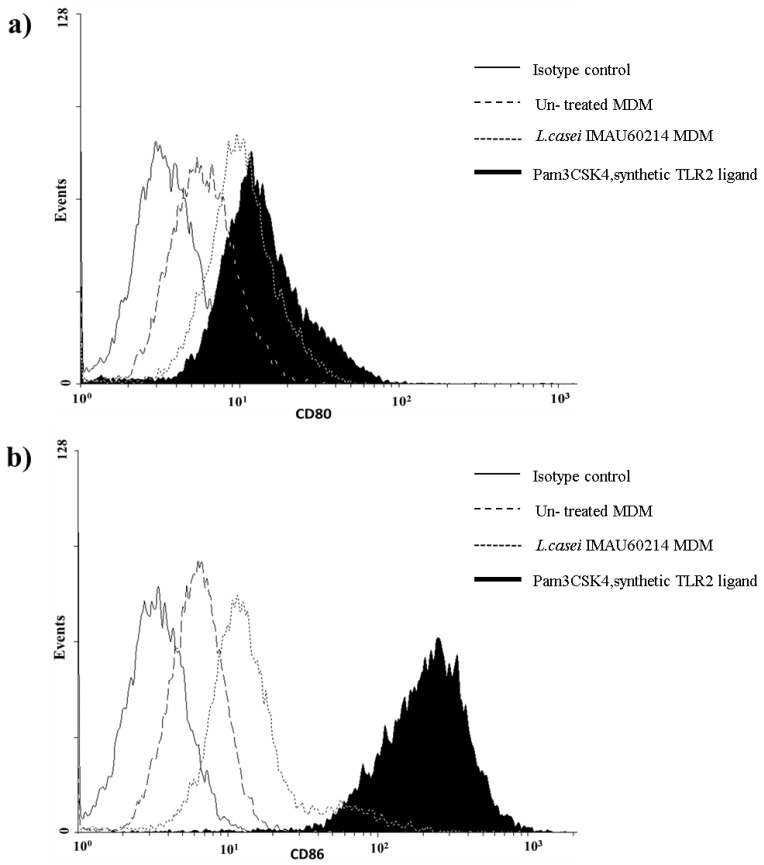
Heat-killed *L. casei* IMAU60214 increases surface expression of CD80 and CD86 by MDMs. Macrophages were cultured in medium alone, 1 µg/mL of the Toll-like receptor 2 (TLR2) agonist Pam3CSK4 (positive control), or *L. casei* IMAU60214 at a ratio (500:1) for 24 h. Cells were then fixed and stained with isotype control, FITC-labeled anti-CD80, or PE-labeled anti-CD86. (**a**) CD80 and (**b**) CD86.

An increase in basal CD80 expression was induced when the MDMs were exposed to *L. casei* IMAU60214 at a ratio (bacteria/MDM) of 500:1 as compared with control cells without stimulation (Figure 4a). In addition, the treatment also increased surface expression of the costimulatory molecule CD86 compared to baseline and negative control stimulation (Figure 4b). Similar results were observed when the MDMs were stimulated with TLR2 agonist (Pam3CSK4) utilized as positive control of the assay, which induced expression substantially greater than that produced by the probiotic heat-killed bacteria.

### 3.5. Induction of Macrophage Phagocytic Activity by Heat-Killed L. casei IMAU60214

**Figure 5:**
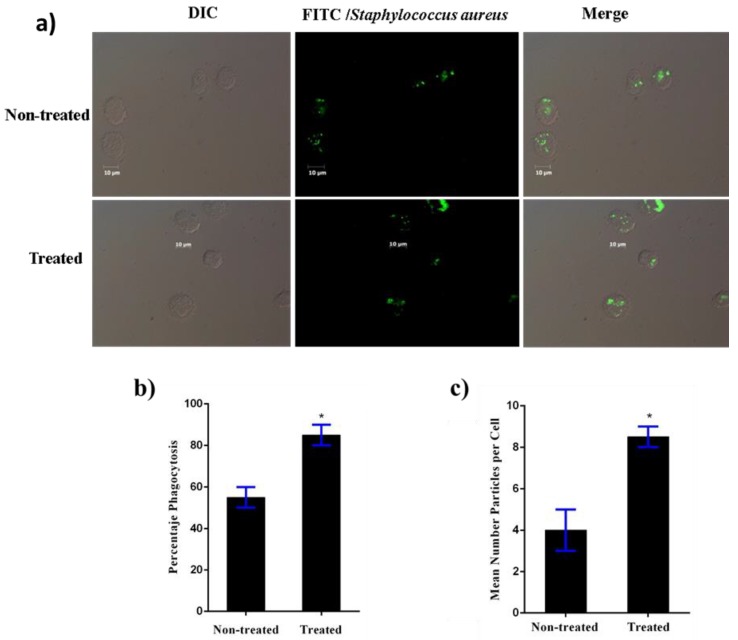
Heat-killed *L. casei* IMAU60214 increases the phagocytosis of *Staphylococcus aureus* by MDMs. Phagocytosis was evaluated by counting phagocytic macrophages, and the number of engulfed particles in randomly selected fields of untreated and treated MDMs with *L. casei* IMAU60214 (at a ratio of 500:1 for 24 h) was challenged with FITC *S. aureus* at a ratio 10:1 (bacteria/MDM). (**a**) Differential contrast (DIC) micrograph showing FITC-*S. aureus* fluorescence in MDMs. (**b**) Percentage of phagocytosis. (**c**) Mean number of particles phagocytized per cell. * A *p* < 0.05 was considered for statistical significance compared to untreated controls for percentage phagocytosis and mean number particles per cells, respectively.

The effect of *L. casei* IMAU60214 at ratio (500:1) on macrophage phagocytosis was investigated by uptake of fluorophore-tagged *Staphylococcus aureus* particles (Figure 5a). Indeed, the number of fluorescent macrophages (containing *S. aureus* particles) increased and the phagocytic index was significantly higher than control (untreated) MDMs (Figure 5b). Thus, exposure to heat-sensitive *L. casei* IMAU60214 promotes the phagocytic activity of macrophages (Figure 5c).

### 3.6. Effects of L. casei IMAU60214 on Macrophages Are Dependent on Activation of Toll-Like Receptor 2

**Figure 6:**
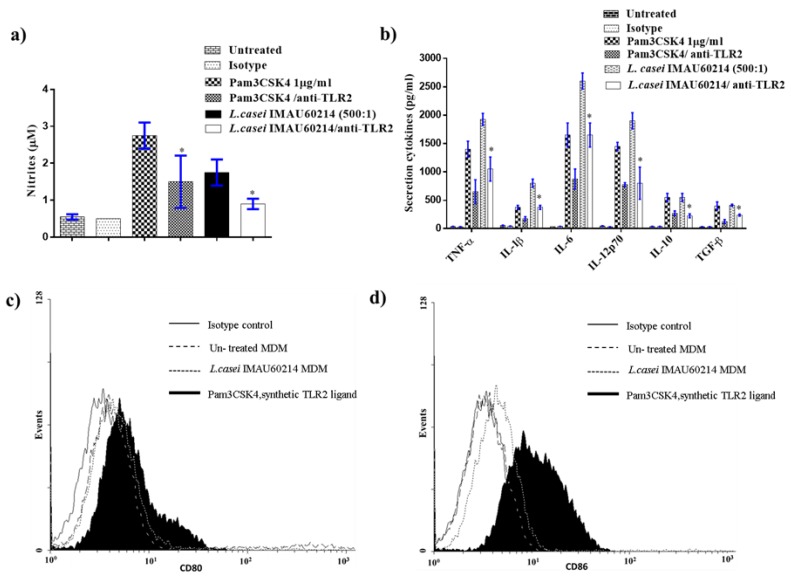
Effects of heat-killed *L. casei* IMAU60214 on MDMs are dependent on the activation of Toll-like receptor 2. MDMs were pre-incubated with anti-TLR2 (10 μg/ml) for 1 h prior to stimulation by *L. casei* IMAU60214 (at ratio of 500:1) or Pam3CSK4 (1 μg) as a positive control for 24 h. Control MDMs were left untreated as a negative control. (**a**) NO accumulation. (**b**) Cytokine production. * Statistically significant *p* < 0.05 difference between conditions. (**c**) CD80 and (**d**) CD86.

As shown (Figure 6), the pretreatment with neutralizing antibody for 1 h significantly reduced NO release by 70% (Figure 6a), and TNF-α, IL-1β, IL-6, IL-12p70, IL-10, and TGF-β release (by 50–70%) (Figure 6b). Similarly, data were observed on surface expression of CD80 and CD86 (Figure 6c,d).

## 4. Discussion

The maintenance of gastrointestinal homeostasis is critical for the prevention of inflammatory and metabolic disorders [30]. The positive influences of probiotics on intestinal homeostasis and host health depend mainly on antagonism of undesirable bacteria and maintenance of a “physiological state of inflammation” that contributes to the control of certain infectious and immunological reactions [31,32]. However, unresolved questions concern the scopes of local and systemic effects as well as the molecular pathways responsible for interactions with host cells. The local immunomodulatory contributions of LAB have been demonstrated in several intestinal mucosal epithelial cell lines and mucosal immune cells (e.g., T lymphocytes, dendritic cells, and monocytes-macrophages) [33,34].

Probiotics have been used as alternative therapies in intestinal inflammatory disorders. It is recognized that the stimulatory and inhibitory actions of probiotic bacteria in the gut are dependent on species, cell condition (i.e., whether viable or dead), and the host-specific effects of soluble components [35,36]. However, there is an increasing interest in the use of non-viable bacteria to reduce the risk of microbial translocation and infection and the possibility of application in some immunocompromised patients (i.e., oncology where the administration of live microorganisms may not be appropriate). In this study, we showed that the probiotic strain *L. casei* IMAU60214 in the non-viable state has immunostimulating activity on human MDMs.

In the present study, we focused on the effects of non-viable bacteria only, because many of the effects obtained from viable cells of probiotics are also obtained from populations of dead cells [37,38]. In addition, the use of dead probiotics as biological response modifiers has several attractive advantages; such products would be very safe and have a long shelf-life. We found that non-viable *L. casei* IMAU60214 enhanced the production of nitric oxide, a mediator of oxygen-dependent microbicide critical for the control of pathogens [39]. Nitric oxide also plays an important role in innate immunity mediated by macrophages. Release of NO can produce cytotoxic reactive species such as peroxynitrites, which are effective antimicrobials [40,41]. The synthesis of NO by MDMs was dependent on the relative abundance of added probiotic bacteria. Moreover, polymyxin B (an LPS inhibitor) effectively inhibited the NO production induced by LPS but had no effect on Pam3CSK4 -induced NO production, indicating that enhanced NO production by *L. casei* IMAU60214 was not the result of endotoxin contamination. Similarly, *L. casei* IMAU60214 also dose-dependently induced the release of both pro- and anti-inflammatory cytokines. TNF-α, IL-1β, IL-6, and IL-12 are inflammatory cytokines that participate in pathogen clearance [42]. TNF-α activates inflammatory cells such as neutrophils, monocytes, macrophages, and dendritic cells, and increases the recruitment of these cells to the site of inflammation. The cytokine TNF-α also promotes the expression of inducible nitric oxide synthase (iNOS), thereby enhancing NO production. Similarly, IL-6 also maintains host immunity as deficiency leads to failure of both innate and adaptive immune responses to viral and bacterial infections [43]. Likewise, IL-12 can activate natural killer cells and stimulate the synthesis of IFN-γ, one of the main activators of macrophages. IL-12 increases the expression of enzymes (NADPH oxidase, iNOS) and also promotes the secretion of cytokines from TH1 cells. In the current study, *L. casei* IMAU60214 activated the release of all of these cytokines from macrophages. Based on the substantial increase in IL-1β production, we suggest that these probiotic bacteria can effectively activate the entire inflammasome, in accordance with previous studies on other *Lactobacillus* strains such as *L. rhamnosus*, demonstrating activation of the inflammasome and suppression of viral infection [44].

On the other hand, *L. casei* IMAU60214 also induced the secretion of anti-inflammatory cytokines such as IL-10 and TGF-β. IL-10 is one of the most important immunosuppressive cytokines for the maintenance of immune homeostasis [45], whereas TGF-β exerts systemic immunosuppression and inhibits host immunosurveillance [46]. Our current findings of enhanced NO and cytokine production are in accordance with a previous report by Jung et al. [47] demonstrating similar effects of *Lactobacillus sakei* KO40706 on RAW264.7 macrophages and the enhancement of NO and cytokine secretion (mainly TNF-α and IL-6) in mice immunosuppressed by cyclophosphamide treatment. Similarly, several strains of *Lactobacillus* have been shown to trigger the release of TNF-α, IL-6, and IL-10 from macrophages and dendritic cells [48]. The efficacy of probiotics to enhance the production of cytokines such as IL-12 and IL-10 is considered a critical attribute for health promotion and possible therapeutic application. In addition, previous research has also demonstrated the capacity of probiotic bacteria to flexibly regulate cytokine production by macrophages and T cells for immune regulation and multifunctional immune activities [49].

In contrast, live and heat-killed *L. casei* Lbs2 (MTCC5953) significantly decreased LPS-induced production of TNF-α and IL-6, and increased the expression levels of IL-10 and TGF-β in mouse colon tissue [50]. Sun et al. [51] also reported downregulation of LPS-induced TNF-α and IL-6 production in mononuclear cells and human THP-1 monocytes by *L. paracasei*. These variations in macrophage effector responses may be related to the different host cells or *Lactobacillus* strains used. In addition to triggering NO and cytokine production by macrophages, *L. casei* IMAU60214 also enhanced the surface expression of the costimulatory molecules CD80 and CD86. Both CD80 and CD86 are generally expressed at low levels, but expression levels are increased upon stimulation by certain molecules such as LPS or Pam3CSK4. In this study, heat-killed *L. casei* IMAU60214 had similar effects. Molecules CD80 and CD86 are the secondary signal of costimulation in antigen-presenting cells and promote the differentiation and maturation of these cells. Moreover, they stimulate the activation of TH1 cells. Increased CD86 and CD80 expression suggests M1 differentiation. In accordance with this finding, a recent study found that *L. johnsonni* NBRC13952 activated IL-1β secretion as well as CD80 expression and that these responses were important for suppression of *Aggregatibacter actinomycetemcomitans* infection [52].

Other studies have reported rapid upregulation of immune-related genes such as IL-7R, CD80, CD86, NF-kappa B, and CCL4 in THP-1 cells upon stimulation by *L. acidophilus* L-92 [53]. Similarly, recent studies have provided evidence that certain *Lactobacillus* species such as *L. crispatus* can increase expression of costimulatory molecules, including CD40, CD80, and CD86, suggesting differentiation of monocytes into precursors of Langerhans-like cells [54]. In the current study, we also demonstrated that heat-killed *L. casei* IMAU60214 exposure can enhance phagocytosis of *Staphylococcus aureus* by MDMs. This is an essential activity of polymorphonuclear cells and monocytes-macrophages in innate immunity. Similar results have been reported using different labeled bacteria as well as *Escherichia coli* [55]. A previous study also documented a significant increase in phagocytosis after treatment with different strains of *Lactobacillus* both in vitro and in vivo [56,57,58].

It is recognized that cellular components derived from probiotics interact via PRRs, including NOD-like receptors and the family of TLRs, which can modulate cytokine production [59]. It has been reported that TLR2 is involved in the secretion of both pro- and anti-inflammatory cytokines in response to probiotic strains [60,61]. We demonstrated that the mechanism responsible for macrophage activation by heat-killed probiotic bacteria is TLR2-dependent, blocking antibody treatments and showing that *L. casei* IMAU60214 acted as a TLR2 agonist in macrophages, in accordance with a recent investigation demonstrating immune regulation through TLR2/TLR6 signaling in response to LAB [62].

Based on these findings, heat-killed *L. casei* IMAU60214 appears to stimulate immune activity by inducing differentiation of macrophages towards the M1 phenotype, which could have immunoregulatory actions in immunocompromised hosts. Additional studies in vivo are warranted to examine the potential of this probiotic strain for improvement of human health.

## 5. Conclusions

In conclusion, this study demonstrates that heat-killed *Lactobacillus casei* IMAU60214 dose-dependently promotes the immune activities of macrophages via TLR2 activation and so may be one of the main routes of activation in early immunity. Likewise, our findings confirm that probiotics in their non-viable condition are potentially beneficial to human health.
